# Hierarchical Porous and Three-Dimensional MXene/SiO_2_ Hybrid Aerogel through a Sol-Gel Approach for Lithium–Sulfur Batteries

**DOI:** 10.3390/molecules27207073

**Published:** 2022-10-20

**Authors:** Jianping Zhou, Ziyuan Pei, Zhuyin Sui, Ying Liang, Xiufeng Xu, Yongpeng Li, Yulin Li, Jingyi Qiu, Qi Chen

**Affiliations:** 1 School of Chemistry & Chemical Engineering, Yantai University, Yantai 264005, China; 2 State Key Laboratory of Marine Resource Utilization in South China Sea, Hainan Provincial Key Lab of Fine Chemistry, School of Chemical Engineering and Technology, Hainan University, Haikou 570228, China; 3 Research Institute of Chemical Defense, Beijing 100191, China

**Keywords:** MXene, SiO_2_, hybrid aerogel, porous structure, lithium–sulfur battery

## Abstract

A unique porous material, namely, MXene/SiO_2_ hybrid aerogel, with a high surface area, was prepared via sol-gel and freeze-drying methods. The hierarchical porous hybrid aerogel possesses a three-dimensional integrated network structure of SiO_2_ cross-link with two-dimensional MXene; it is used not only as a scaffold to prepare sulfur-based cathode material, but also as an efficient functional separator to block the polysulfides shuttle. MXene/SiO_2_ hybrid aerogel as sulfur carrier exhibits good electrochemical performance, such as high discharge capacities (1007 mAh g^–1^ at 0.1 C) and stable cycling performance (823 mA h g^–1^ over 200 cycles at 0.5 C). Furthermore, the battery assembled with hybrid aerogel-modified separator remains at 623 mA h g^–1^ over 200 cycles at 0.5 C based on the conductive porous framework and abundant functional groups in hybrid aerogel. This work might provide further impetus to explore other applications of MXene-based composite aerogel.

## 1. Introduction

With the development of electronic devices and electric vehicles, the demand for energy-efficient and cost-effective secondary batteries is growing, which has firmly drawn considerable attention and captured the heart of researchers [1]. Among energy storage applications, lithium–sulfur batteries (LSBs), as the next-generation inspiring devices, have shown the attractive potential by virtue of high energy density (up to 2600 Wh kg^–1^), environmental benignancy, and reduced cost [2]. Although significant progress has been made, its large-scale implementation still faces many problems, in particular, mechanical degradation of the cathode because of the huge volume change (nearly 80%) in the process of discharging and charging, the inherent insulation property of sulfur, and the loss of active substances (resulting in shuttle effect of soluble polysulfides) [3,4]. The main obstacle behind the poor battery performance is the dissolution and “shuttle effect” of intermediate polysulfides, which begins with the battery discharge process [5,6].

In order to solve the above problems related to LSBs, progress has been made in various sulfur hosts to block the diffusion and shuttle of polysulfides, so as to obtain more efficient LSBs [7,8]. Different materials with abundant adsorption sites, such as conductive polymers [9,10], porous carbon nanofibers [11], hierarchical porous carbon [12], carbon nanotubes [13], and metal oxides [14,15], are designed to capture active materials to deal with polysulfide shuttle effect, improve battery discharge capacity, and enhance cycling stability. Previous studies have shown these methods can increase capacity and improve the cyclability, but there is still a large amount of loss of active substances. The main reason is that there is no strong adsorption potential, which can not only adsorb polar polysulfides but also solve the loss of active substances.

The continuous progress in research and development of functional separators has significantly improved the cycling performance of LSBs [16,17]. The functional separator is easy to prepare, and it can be used as an effective barrier to inhibit the shuttle effect of polar polysulfides across separators, and as a reservoir for different sulfur species [18,19]. Different types of modified separators, such as the dual-doped mesoporous carbon-modified separator [20], titanium dioxide-modified separator [21], and metal–organic framework-based separator [22], have been prepared to limit the movement of polysulfide intermediates to the cathode. The use of two-dimensional materials in functional separators of LSBs has aroused a great interest of researchers, owing to their large specific surface area and rich functional groups. For example, Huang’s team prepared a permselective graphene oxide-modified separator, which allows lithium ions to penetrate, but repels anions. The resultant separator can adequately block the shuttle of polysulfides through steric exclusion and electrostatic repulsion, and the corresponding capacity loss rate of the battery with the modified separator was 0.23% per cycle [23]. To overcome the challenge of polysulfide diffusion, Cui and his colleagues prepared a bifunctional separator with two-dimensional black-phosphorus nanosheets, which can capture different polysulfides through the strong interaction and re-activation of captured polysulfides owing to its high lithium ion diffusivity and electron conductivity [24].

MXene obtained from MAX phase materials is a new kind of two-dimensional transition layered metal carbides/nitrides/carbonitrides [25,26]. MXene exhibits great potential in LSBs because of its rich surface functional groups and excellent conductivity, which play important roles in immobilizing soluble polysulfides [27,28]. However, because of hydrogen bonding interactions and van der Waals force, MXene sheets tend to restack, which will inevitably result in the loss of the active sites and limit the adsorption and catalytic conversion of polar polysulfides. Therefore, it is essential to alleviate the aggregation of MXenes and assemble MXenes into three-dimensional (3D) porous materials, so as to better promote the effective adsorption/conversion of polysulfides and improve the battery performance.

Recently, SiO_2_ has been developed as a sulfur host material or functional separator for LSBs. For example, Kou et al. reported a flexible carbon/SiO_2_ membrane material through a phase inversion method for LSBs. The as-designed carbon/SiO_2_-derived sulfur-based cathodes presented long-term cycling stability [29]. Li et al. prepared a SiO_2_ particle-modified polypropylene (PP) separator by immersing the PP membrane in the hydrolysis solution of tetraethyl orthosilicate (TEOS). The rate capability and cyclic stability of the LSBs using the SiO_2_-modified separator were greatly improved [30]. However, the work on MXene/SiO_2_-based aerogel materials for high-performance LSBs has never been reported.

Herein, we report a design of a unique porous material, namely, MXene/SiO_2_ hybrid aerogel (MSHA) with a high specific surface area value (572 m^2^ g^–1^) via facile sol-gel and freeze-drying methods. The hierarchical porous hybrid aerogel can be used not only as a novel host for the sulfur-based cathode but also as an efficient functional separator to alleviate the shuttle effect of polysulfides. MSHA as a sulfur carrier exhibits high discharge capacities, excellent rate capability, and good cycling performance. MXene sheets can help to provide a conductive network for rapid electron transfer between the collector and active substances. It is worth noting that the SiO_2_ components are used as pillars to support MXene sheets to construct open spaces. Furthermore, the battery containing MSHA-functionalized separator shows high capacities (623 mA h g^–1^ over 200 cycles at 0.5 C). We believe that this work will provide further impetus for the researchers to explore applications of MXene-based composite aerogel in LSBs.

## 2. Results and Discussion

### 2.1. Preparation of MSHA

**Figure 1:**
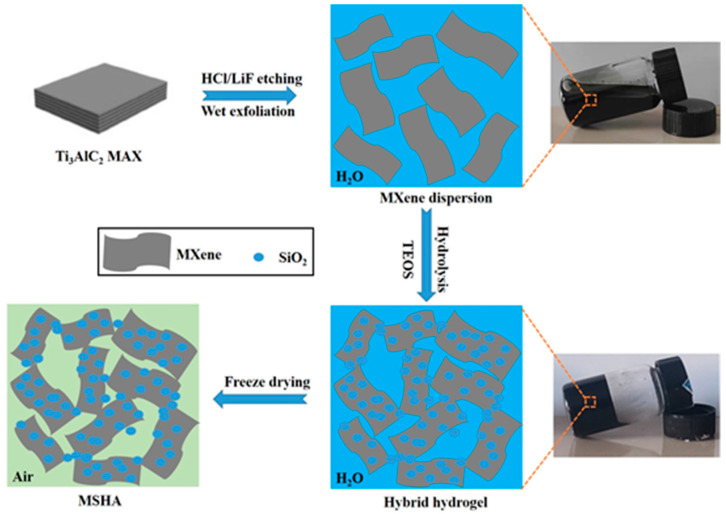
The preparation diagram of MSHA.

Figure 1 shows the preparation process of MSHA. It starts with the employment of aqueous Ti_3_C_2_T_x_ MXene solution as the precursor. MXene (Ti_3_C_2_T_x_) sheets were prepared by selective removal of Al from Ti_3_AlC_2_ MAX material with a wet etching (HCl + LiF) system [31]. Transmission electron microscopy analysis (Figure S1) illustrates the two-dimensional nature of MXene sheets. MXene sheets can be applied to prepare porous materials by assembling different materials through sol–gel and freeze-drying methods [32]. After TEOS was added into MXene aqueous dispersion, TEOS can be converted into SiO_2_ by hydrolysis and polymerization reaction [33]. The hydroxyl functional groups on the surface of SiO_2_ can form hydrogen bond with oxygenated functional groups of Ti_3_C_2_T_x_ MXene. As the reaction proceeded, MXene/SiO_2_ hybrid hydrogel was obtained. The SiO_2_ formed in situ is randomly mixed with MXene. In addition, in order to have a good understanding of the hybrid hydrogel, the rheological behavior of the as-prepared hybrid hydrogel was studied using oscillatory rheometry at room temperature (Figure S2). The formation of crosslinking network can be confirmed by the higher storage modulus G’ [34], which is eight times larger than the loss modulus G” at the angular frequency of 10 rad/s. Finally, MSHA was prepared through freeze-drying process.

### 2.2. Microstructures of MSHA and Ti_3_C_2_T_x_ MXene

**Figure 2:**
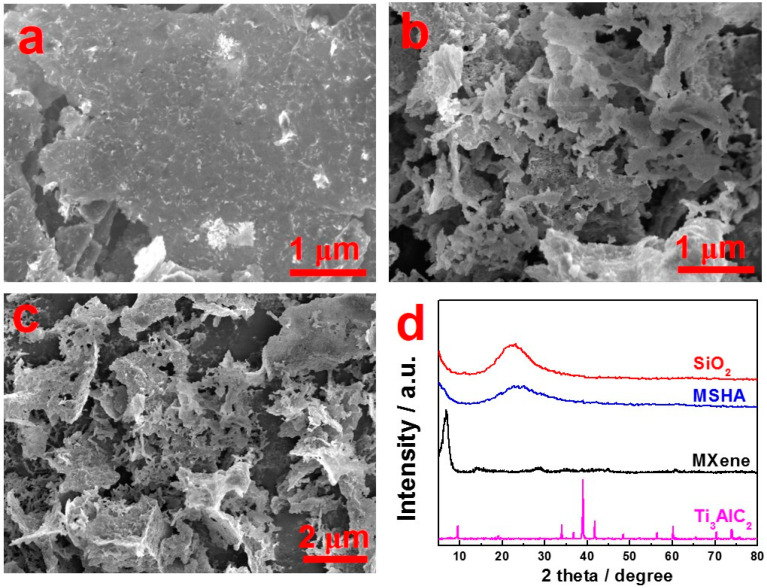
SEM images of the etched Ti_3_C_2_T_x_ sample (**a**) and porous MSHA (**b**,**c**); (**d**) XRD patterns of Ti_3_AlC_2_, SiO_2_, MSHA, and Ti_3_C_2_Tx.

The morphology of MSHA and Ti_3_C_2_T_x_ MXene samples was analyzed by scanning electron microscope (SEM). Ti_3_C_2_T_x_ MXene (Figure 2a) presents a dense plane structure, indicating that MXene sheets are easy to stack closely together. After the hydrolysis and polymerization of TEOS on the MXene surface, the as-prepared MSHA (Figure 2b–d) displays a crumbled 3D porous network. Furthermore, uniform Ti, O, Si, and C elemental distribution images (Figure S3) were observed from the energy-dispersive spectroscopy mapping. The SiO_2_ particles should be randomly mixed with MXene.

The phase transition of raw Ti_3_AlC_2_, Ti_3_C_2_Tx, SiO_2_, and MSHA was studied by X-ray diffractometer (Figure 2d). The structural evolution of Ti_3_C_2_T_x_ and Ti_3_AlC_2_ was revealed by comparing their X-ray diffraction (XRD) data. The XRD pattern data of Ti_3_AlC_2_ presents diffraction peaks at 2θ = 9.5°, 19.1°, 34.0°, 38.9°, and 41.7°, which can be ascribed to the contribution from (002), (004), (101), (104), and (105) crystal planes. The strongest (104) peak disappears completely due to the LiF/HCl etching treatment. At the same time, the (002) basal plane peak shifts from 9.5° (Ti_3_AlC_2_) to 6.9° (Ti_3_C_2_T_x_ MXene), which is consistent with the reported literature [35,36]. The movement of (002) peak to a relatively lower angle suggests an increase in the spacing of layers. This is due to the removal of element Al in Ti_3_AlC_2_ and the introduction of surface termination functional groups (–F, –O, and –OH) in Ti_3_C_2_T_x_. For MSHA sample, the peak at 6.9° attributed to the (002) plane of MXene is less pronounced, whereas a new wide peak located at around 23° appears, indicating the successful introduction of amorphous SiO_2_.

**Figure 3:**
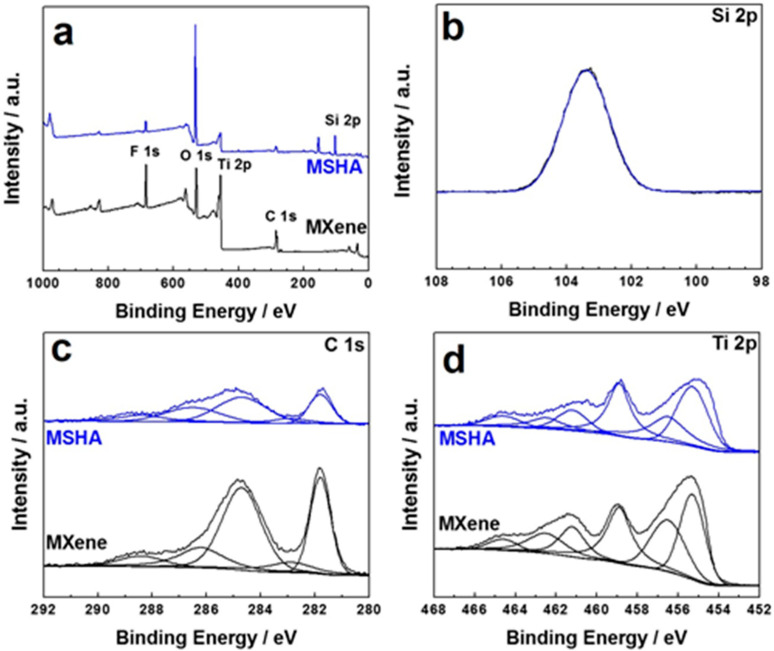
XPS spectra of the as-prepared samples: (**a**) full survey spectra, (**b**) Si 2p spectrum of MSHA, (**c**) C 1s, and (**d**) Ti 2p spectra.

X-ray photoelectron spectroscopy (XPS) characterization has been carried out to elucidate the chemical elements and their chemical states. Figure 3a indicates that MXene possesses C, O, F, and Ti elements. There is an additional Si element for MSHA after the introduction of SiO_2_. The Si 2p spectrum (Figure 3b) of MSHA is composed of a SiO_2_ peak (103.4 eV), giving evidence to the existence of SiO_2_ [37]. As shown in Figure 3c, the C 1s spectra has been divided into four chemical states, including C–Ti (281.8 and 282.9 eV), graphitic C–C (284.7 eV), C–O (286.2 eV), and C=O (288.4 eV), respectively. As displayed in Figure 3d, the Ti 2p spectra has been divided into six chemical states, including Ti–C (455.3 eV), Ti 2p3/2 signal (456.5 eV), Ti–OH (458.9 eV), Ti^2+^ (461.2 eV), Ti 2p1/2 signal (462.5 eV), and Ti–O_x_ (464.6 eV), respectively. The O 1s XPS spectra of Ti_3_C_2_T_x_ MXene and MSHA can be found in Figure S4, and their O 1s peaks possess two peaks located at 529.9 and 531.8 eV, which might be attributed to C–Ti–O_x_ and Ti–OH signals, respectively. For MSHA, a new peak centered at 532.5 eV appears (Figure S4), which can be indexed to Si–O group of SiO_2_ [37].

**Figure 4:**
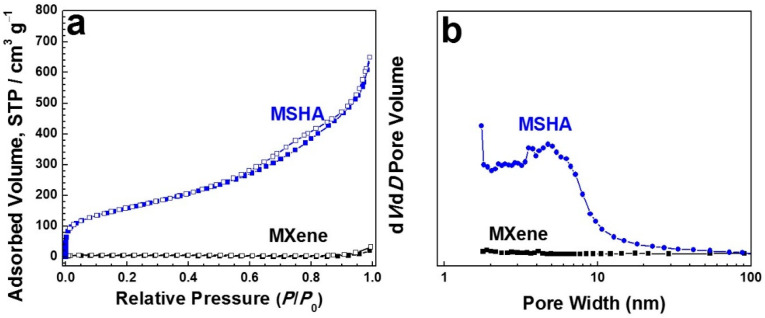
Porous property of the obtained MSHA and MXene: (**a**) N_2_ sorption isotherms and (**b**) pore size distributions.

The textural properties of the as-prepared MSHA and MXene were illustrated by N_2_ sorption analysis. The N_2_ sorption isotherm (Figure 4a) of MSHA shows a type-IV isotherm along with a type-H3 hysteresis loop at the high pressure range, indicating the presence of mesopores and macropores. The volume adsorbed at low-pressure range increases sharply, suggesting the existence of micropores [38]. Therefore, the as-prepared hybrid aerogel possesses a hierarchically porous structure. The surface area and pore volume values of the obtained MSHA are 572 m^2^ g^–1^ and 1.0 cm^3^ g^–1^, respectively, which are larger than those of Ti_3_C_2_T_x_ MXene (13 m^2^ g^–1^ and 0.013 cm^3^ g^–1^), indicating that the porosity properties were largely enhanced after the introduction of SiO_2_. As displayed in Figure 4b, MSHA shows a wide pore size distribution in the size range of 1–30 nm, while Ti_3_C_2_T_x_ lacks these pores due to the severe stacking of MXene sheets. The micro–meso–macro porous structure of MSHA might be conducive to improving the utilization of sulfur-based cathodes. Furthermore, the porous architecture with adsorption site is also conducive to preventing the shuttle of polysulfides [39].

### 2.3. Battery Performance of MSHA as Sulfur Host

**Figure 5:**
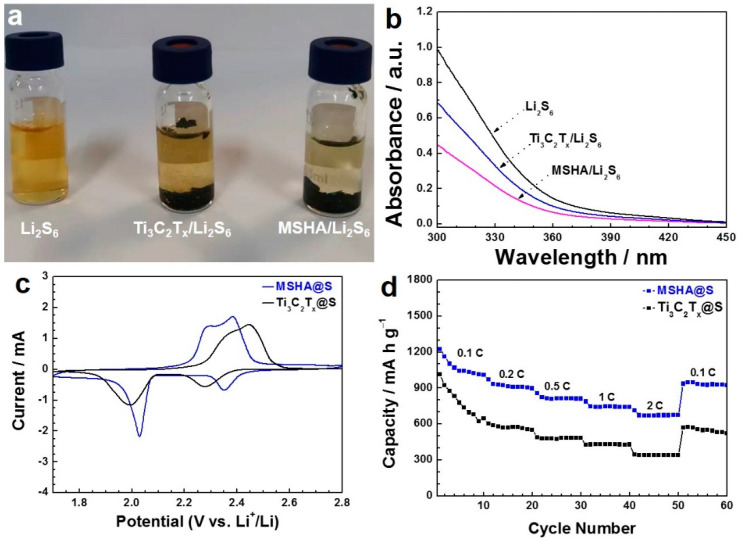
(**a**) The visual observation on the interaction between the as-prepared samples and polysulfides; (**b**) the ultraviolet–visible absorption spectra of L_2_S_6_ solution in (**a**); (**c**) cyclic voltammogram curves of Ti_3_C_2_T_x_@S and MSHA@S cathodes at 0.1 mV/s; (**d**) rate performance of MSHA@S and Ti_3_C_2_T_x_@S.

MSHA possesses good potential as sulfur host because of its high porosity, stable chemical structure, and polar framework. It has been reported that SiO_2_ particles can interact with polysulfides [40]. During charge/discharge process, soluble lithium polysulfides can be formed. Li_2_S_6_ is one of soluble lithium polysulfides. In our work, the interaction of SiO_2_ components and Li_2_S_6_ was identified by visual observation. As displayed in Figure 5a, MSHA and MXene were added to Li_2_S_6_ solution. The color of the solution including MSHA was lighter, compared to MXene, because of the strong adsorption of SiO_2_ to polysulfides. Figure 5b presents their corresponding ultraviolet–visible absorption spectra, further confirming the good adsorption capacity of MSHA for Li_2_S_6_.

In order to evaluate the electrochemical performance of the as-prepared samples as sulfur hosts, MSHA@S and Ti_3_C_2_T_x_@S were prepared, according to a sulfur impregnation method [41]. A series of electrochemical tests were used to study the electrochemical performance of MSHA@S (or Ti_3_C_2_T_x_@S). The battery was fabricated in a glovebox by employing MSHA@S (or Ti_3_C_2_T_x_@S) as cathode material, metallic lithium as anode, and PP as separator. The cyclic voltammetry was carried out in the potential range of 1.7–2.8 V at 0.1 mV s^–1^ (Figure 5c). There are two strong peaks at about 2.35 and 2.02 V in the cathodic scanning of MSHA@S cathode. The former can be ascribed to the chemical transition from S_8_ to soluble polysulfides (Li_2_S_x_, 4 ≤ x ≤ 8), and the latter is related to the further chemical reduction of high-order polysulfides to low-order Li_2_S_2_ and Li_2_S [42,43]. For the anodic scanning process, there are two peaks at around 2.28 and 2.38 V, which correspond to the chemical reaction of Li_2_S to polysulfides, and subsequently to S_8_ [44,45].

**Figure 6:**
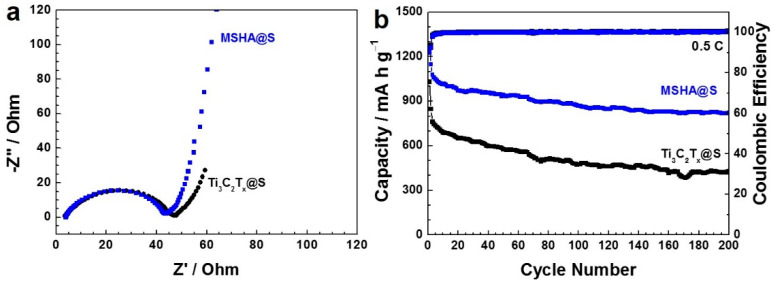
(**a**) Electrochemical impedance spectroscopy curves and (**b**) cycling stability (0.5 C) of the batteries with MSHA@S and Ti_3_C_2_T_x_@S cathodes.

The charge/discharge experiment was conducted at each current density for ten cycles (Figure 5d), and the corresponding experimental data were shown in Figure S5. MSHA@S cathode showed a discharge capacity of 1007 mA h g^–1^ (cycle 10) at 0.1 C, 897 mA h g^–1^ (cycle 20) at 0.2 C, and 811 mA h g^–1^ (cycle 30) at 0.5 C, respectively. Even at 1 and 2 C, MSHA@S still presented discharge capacities of 739 mA h g^–1^ (cycle 40) and 673 mA h g^–1^ (cycle 50), respectively. In particular, a discharge capacity of 923 mA h g^–1^ (cycle 60) was recovered when the current density returned to 0.1 C, suggesting the excellent tolerance and good stability of MSHA@S. Obviously, the reversible discharge capacities of MSHA@S cathode were higher than those of Ti_3_C_2_T_x_@S cathode. Electrochemical impedance spectroscopy experiments were conducted to acquire more information on MSHA@S and Ti_3_C_2_T_x_@S cathodes. The data were displayed in Figure 6a. The semicircle at high-frequency region and the inclined line at low-frequency region stand for the charge transfer resistance and the ion diffusion resistance, respectively [46]. MSHA@S cathode presents a smaller charge transfer resistance than Ti_3_C_2_T_x_@S cathode, indicating that MSHA could promote electron transport and facilitate ion migration.

The cycling performance of the battery with MSHA@S cathode was conducted for 200 cycles at 0.5 C (Figure 6b and Figure S6). The assembled battery was activated for two cycles at low current density prior to cycle tests. Apparent from the data, the battery presented an initial specific capacity of 1078 mA h g^–1^ and maintained 823 mA h g^–1^ even after 200 cycles at 0.5 C. However, a low discharge capacity (429 mA h g^–1^) was observed for the battery with Ti_3_C_2_T_x_@S cathode. In addition, MSHA@S cathode was superior to many other SiO_2_-based cathodes, such as SiO_2_-coated sulfur particles (763.2 mA h g^–1^ at 0.1 C after 50 cycles) and sulfur/SiO_2_/partially reduced graphene oxide (491 mA h g^–1^ at 0.2 C after 300 cycles) [47,48]. The improved electrochemical properties might be attributed to hierarchically porous network structure and strong interaction between SiO_2_ components and polysulfide species. In particular, the synergetic effect of hierarchically micro–meso–macro pore structures plays a vital role: the macroporous structure promotes electrolyte penetration, mesopores provide a way for ion transport and electrolyte diffusion, and the microporous structure serving as a reservoir can inhibit the dissolution of lithium polysulfide species, thus, preventing the shuttle effect [49]. Furthermore, the porous architecture is conducive to buffering volume expansion of sulfur particles during discharge.

### 2.4. Battery Performance of Modified Separators

Prior to battery performance tests of modified separators, the polysulfide diffusion through the as-prepared separators was studied by H-shaped permeating experiment. It can be observed from Figure S7 that the color of the electrolyte employing PP is darker than that employing MSHA/PP after 32 h, indicating the MSHA/PP separator could alleviate polysulfide shuttle.

**Figure 7:**
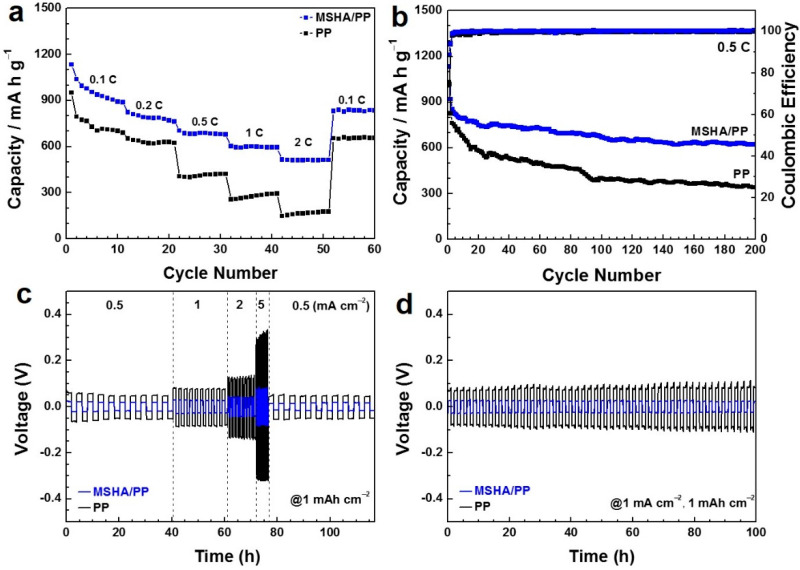
(**a**) Rate capability and (**b**) cycling stability of the cells configured with MSHA/PP and PP; (**c**) rate capability, and (**d**) cycling performance of Li||Li symmetric cells configured with MSHA/PP and PP.

Carbon black (CB) was usually applied as a sulfur host to assess the battery performance of modified separator [50]. Therefore, coin cells were assembled based on the S/CB cathode, lithium anode, and the MSHA-functionalized separator. The discharge/charge data of the cell configured with MSHA/PP were shown in Figure S8. There were two charge plateaus and two discharge plateaus in these curves. The discharge plateaus are related to the chemical transition from S_8_ to soluble polysulfide species (Li_2_S_x_, 4 ≤ x ≤ 8) and further to Li_2_S_2_ and Li_2_S. The charge plateaus stand for the chemical oxidation of Li_2_S to polysulfides and eventually to S_8_ [42]. It can be observed from experimental data that the plateaus of the cell with MSHA/PP are obvious at 2.0 C. It could be deduced accordingly that the MSHA/PP might effectively prevent the shuttle of polysulfides. The charge/discharge tests of the cell with MSHA/PP were studied at each current density for ten cycles (Figure 7a). It is obvious that the rate capability of the cell with MSHA/PP was superior to that with PP separator, indicating that the MSHA/PP was more beneficial for rapid electron/ion transport and redox reaction kinetics than PP separator.

The cycling performances of the cell with different separators over 200 cycles at 0.5 C were studied (Figure 7b). According to their charge/discharge experimental data (Figure S9), the assembled cell with MSHA/PP also presents two discharge platforms, corresponding to the conversion of S_8_ to polysulfides, and subsequently to Li_2_S_2_/Li_2_S. The cell with MSHA/PP delivers a decent discharge capacity of 623 mA h g^–1^ after 200 cycles at 0.5 C. In contrast, the cell with PP separator shows a low capacity and fast decay (from 762 mA h g^–1^ to 340 mA h g^–1^) during cycling. The above analysis indicates that MSHA/PP plays a vital role in inhibiting polysulfides shuttling and improving redox reaction kinetics. In addition, MSHA/PP separator was also superior to the SiO_2_-based separator previously reported (603.5 mA h g^–1^ at 0.2 C after 200 cycles) [30]. The improved electrochemical performance of the cell with MSHA/PP might be attributed to the following reasons. First, the 3D conductive Ti_3_C_2_T_x_-based framework with porous structure is beneficial for the electron/ion accessibility. Second, the rich functional groups in MSHA are conducive to the adsorption of polysulfides, thus, alleviating the shuttle effect [51]. Furthermore, we also studied the effect of MSHA-modified separator on lithium anode by means of the Li||Li symmetric battery method. As shown in Figure 7c, compared with PP, MSHA/PP presents narrower voltage hysteresis. Figure 7d exhibits cycle performance of Li||Li symmetric batteries configured with MSHA/PP and PP. Obviously, the cell with MSHA/PP displays a low polarization behavior, indicative of the effective suppression of dendrite growth [50].

## 3. Materials and Methods

### 3.1. Preparation of MXene/SiO_2_ Hybrid Aerogel (MSHA) Material

The preparation process of Ti_3_C_2_T_x_ MXene dispersion was provided in the supplementary file. During the preparation of MSHA, TEOS (0.5 mL), ethanol (2 mL), and deionized water (4 mL) were first mixed together. To promote the hydrolysis of TEOS, an appropriate amount of hydrochloric acid was added until the pH value of the mixture became around 4. After stirring at room temperature for 15 min, in order to promote the further condensation of TEOS, an appropriate amount of ammonia was added to the mixture to place its pH near 8. Subsequently, 6 mL of Ti_3_C_2_T_x_ MXene dispersion (10 mg mL^–1^) was added into the above mixture. Afterwards, the mixture was treated at 35 °C for 72 h to form a black hydrogel through a sol-gel method. In the above process, TEOS was simultaneously converted to SiO_2_. MXene/SiO_2_ hybrid aerogel (MSHA) was prepared by purifying the obtained hydrogel with ultrapure water, followed by vacuum freeze-drying of the hybrid hydrogel for 24 h.

### 3.2. Preparation of MSHA@S Composite

First, 40 mg of the hybrid aerogel material was added to the mortar, and then 60 mg of the sublimated sulfur powder was added. After thoroughly grinding the above mixture for 30 min, it was transferred to a horizontal tubular furnace and further heated for 12 h 155 °C. The sample was collected after the tubular furnace was allowed to cool to room temperature. The as-obtained product was marked as MSHA@S. Ti_3_C_2_T_x_@S and S/carbon black (S/CB) composites were synthesized under the same conditions.

### 3.3. Preparation of MSHA Modified Separator

Typically, 80 wt% of the MSHA, 10 wt% of carbon black, and 10 wt% of polyvinylidene fluoride were mixed together and dispersed in a solvent of N-methyl-2-pyrrolidinone. Then, the obtained slurry was coated onto the Celgard2500 PP separator. MSHA modified PP separator (MSHA/PP) can be prepared after drying it at 60 °C overnight.

### 3.4. Material Characterization and Electrochemical Measurement

The information on material characterization and electrochemical measurement can be found in the supplementary file.

## 4. Conclusions

In summary, we report a unique porous material, namely, MSHA with a high specific surface area (572 m^2^ g^–1^) via facile sol-gel and freeze-drying methods. The as-prepared porous material displays a 3D interconnected network structure assembled by Ti_3_C_2_T_x_ MXene and SiO_2_. The hierarchically porous MSHA can be used not only as a new sulfur host to prepare sulfur-based cathode material but also as an efficient functional separator to alleviate the shuttle effect of polysulfides. MSHA as a sulfur carrier exhibits excellent electrochemical performance with a high-rate capability of 673 mA h g^–1^ at 2 C and a good cycling performance of 823 mA h g^–1^ after 200 cycles at 0.5 C. Furthermore, the battery assembled with MSHA-modified separator shows high capacities of 623 mA h g^–1^ after 200 cycles at 0.5 C. This work may promote the development of MXene-based aerogels for high-performance Li-S battery.

## Data Availability

Not applicable.

## Supplementary Materials

The following supporting information can be downloaded at: https://www.mdpi.com/article/10.3390/molecules27207073/s1, Figure S1: Transmission electron microscopy picture of Ti_3_C_2_T_x_ MXene nanosheets; Figure S2: The rheological behavior of the as-prepared hybrid hydrogel; Figure S3: The SEM image of porous MSHA and its corresponding elemental mapping; Figure S4: O 1s XPS spectra of MSHA and Ti_3_C_2_T_x_ MXene; Figure S5: Charge/discharge curves of the cell with the MSHA@S cathode from 0.1 to 2.0 C; Figure S6: Charge/discharge curves of the cell with the MSHA@S cathode at 0.5 C; Figure S7: Visualization of the polysulfide diffusion across MSHA/PP and PP separators; Figure S8: Charge/discharge curves of the cell with MSHA/PP separator from 0.1 to 2.0 C; Figure S9: Charge/discharge curves of the cell with MSHA/PP separator at 0.5 C. Reference [31] is cited in the supplementary materials.
