# Supplementary material for: Hierarchical Porous and Three-Dimensional MXene/SiO2 Hybrid Aerogel through a Sol-Gel Approach for Lithium–Sulfur Batteries
Source: Molecules. 2022 Oct 20;27(20):7073. doi: 10.3390/molecules27207073 (PMC9610511; doi:10.3390/molecules27207073)
Supplement: Supplementary file 1 [file molecules-27-07073-s001.zip › molecules-1957989-supplementary.pdf]

Supplementary Material for

Hierarchical Porous and Three-Dimensional MXene/SiO_2 Hybrid Aerogel through A Sol-Gel Approach for Lithium–Sulfur Batteries

Jianping Zhou 1, Ziyuan Pei 1, Zhuyin Sui 1,*, Ying Liang 2, Xiufeng Xu 1, Yongpeng Li 1, Yulin Li 1, Jingyi Qiu 3,* and Qi Chen 2,*

*****Correspondence: suizy@ytu.edu.cn (Z. Sui); qiujingyi1202@163.com (J. Qiu); chenqi@hainanu.edu.cn (Q. Chen)

S1. Materials

Ti_3AlC_2 MAX phase powders (99 wt %) were purchased from Laizhou Kaixi Ceramic Materials Co., Ltd. Sulfuric acid (98 wt %), hydrochloric acid (HCl, 37 wt %), lithium fluoride (LiF, 99.9 wt %), and ethanol (99.7 wt %) are of analytical grade and were purchased from Beijing Chemical Reagents Company. Tetraethyl orthosilicate (TEOS, 99.9 wt %), polyvinylidene fluoride binder (99.5 wt %), and sublimed sulfur (99.9 wt %) were bought from Sinopharm chemical reagent Co. Ltd, China. Electrolyte (2.0% lithium nitrat and 1.0 M bistrifluoromethanesulfonimide lithium in a mixed solvent in 1,3-dioxalane and 1,2-dimethoxyethane 1:1 (vol %)) aluminum foil, and Celgard 2500 membrane were purchased from Guangdong Canrd New Energy Technology Co., Ltd., China.

S2. Synthesis of $Ti_3C_2T_x$ nanosheets

$Ti_3C_2T_x$ nanosheets were prepared by selective etching of Al from Ti_3AlC_2 MAX phase powders using a LiF/HCl mixture [S1]. First, 1 g of LiF powder was introduced into 20 mL of HCl solution (9 M) and stirred thoroughly for five min to dissolve LiF. Then, 1 g of Ti_3AlC_2 powder was slowly added to the solution, which was stirred for 24 h at 38 °C for etching Al. Next, the mixture was washed with ultra-pure water several times by centrifugation at 3500 rpm until the pH of the supernatant liquid reached to approximately 6. To obtain the $Ti_3C_2T_x$ colloidal suspension, the above solution was sonicated under nitrogen for 2 h and then centrifuged at 3500 rpm for 30 min. Finally, the supernatant was collected and its concentration was known to be about 10 mg/mL by vacuum drying.

S3. Synthesis of the Li_2S_6 solution and visualized adsorption test

Li_2S and sublimed sulfur with moderate molar ratio (1:5) were added into 1,3-dioxalane and 1,2-dimethoxyethane (1:1 v/v), and the Li_2S_6 solution was obtained by magnetic stirring at 50 °C for 72 h. Subsequently, visualized adsorption test was performed by adding MSHA and MXene into Li_2S_6 solution (1.0 mL, 10 mM), respectively. The quantity of MXene and MSHA added was 10 mg.

S4. Characterization methods

The morphology and structure of samples were determined by X-ray diffraction (XRD Bruker AXS D8 diffractometer with Cu Kα radiation), transmission electron microscopy (HT7700), and scanning electron microscopy (JEOL JSM-840), respectively. The energy dispersive spectroscopy attached to the scanning electron microscopy was used to obtain the element mapping image. The Brunauer–Emmett–Teller specific surface area was evaluated based on the obtained nitrogen adsorption–desorption isotherms. The nitrogen sorption isotherms at 77 K were acquired by BSD-660M A6B3M specific surface analyzer (BeiShiDe Instrument, China). X-ray photoelectron spectroscopy was carried out on a Kratos AXIS SUPRA spectrometer. Rheological tests were conducted on a Physica MCR 301 Rheometer with 25 mm diameter parallel-plate geometry at 25 °C.

S5. Electrochemical characterization

In order to evaluate the electrochemical performance of MSHA@S composite, the cathode was made of 80 wt % MSHA@S composite, 10 wt % polyvinylidene fluoride, and 10 wt % carbon black. The mixture containing MSHA@S, polyvinylidene fluoride, and carbon black was stirred in N-methyl pyrrolidone, and then the obtained slurry was coated onto a carbon-coated aluminum foil. Before assembling into a coin cell, the electrode was dried at 50 °C for 12 h under vacuum. 1.0 M LiTFSI and 0.1 M lithium nitrate in a solvent of 1,3-dioxolane and 1,2-dimethoxy ethane (1:1, volume ratio) were used as an electrolyte. Celgard 2400 membrane and lithium foil were used as the separator and the anode, respectively. For comparison, $Ti_3C_2T_x$@S was also used as battery cathode.

In order to evaluate the electrochemical performance of MSHA/PP separator, the cathode was made of 80 wt % S/CB composite, 10 wt % polyvinylidene fluoride, and 10 wt % carbon black. The mixture containing S/CB, polyvinylidene fluoride, and carbon black was stirred in N-methyl pyrrolidone, and then the obtained slurry was coated onto a carbon-coated aluminum foil. Before assembling into a coin cell, the electrode was dried at 50 °C for 12 h under vacuum. 1.0 M LiTFSI and 0.1 M lithium nitrate in a solvent of 1,3-dioxolane and 1,2-dimethoxy ethane (1:1, volume ratio)

were used as an electrolyte. MSHA/PP and lithium foil were used as the separator and the anode, respectively. For comparison, PP was also used as battery separator.

A 2032 coin cell was assembled in a glove box. Cyclic voltammogram curves were recorded by using a CHI 760E workstation (Shanghai Chenhua, China). Galvanostatic charge/discharge tests were carried out on 2001A Land battery test system to assess the rate performance and cycle stability. The electrochemical impedance spectroscopy was also conducted on CHI 760E from 100 kHz to 0.01 Hz.

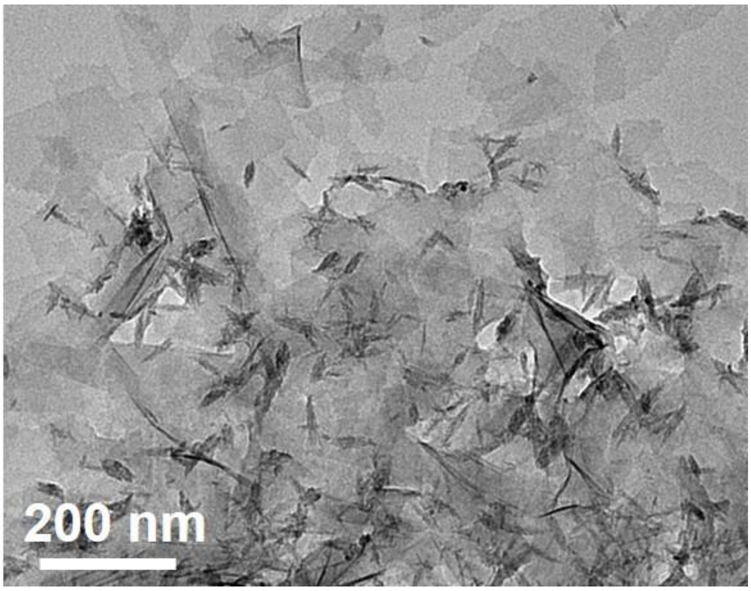

Figure S1. Transmission electron microscopy picture of $Ti_3C_2T_x$ MXene nanosheets.

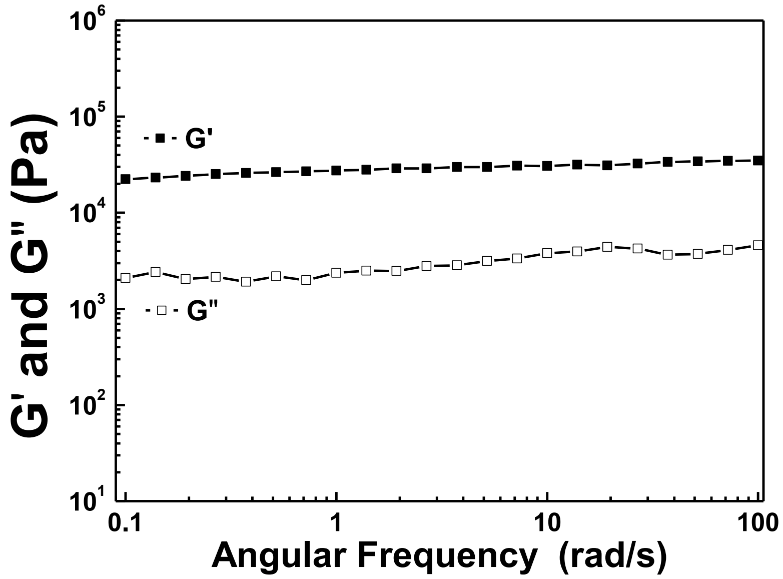

Figure S2. The rheological behavior of the as-prepared hybrid hydrogel.

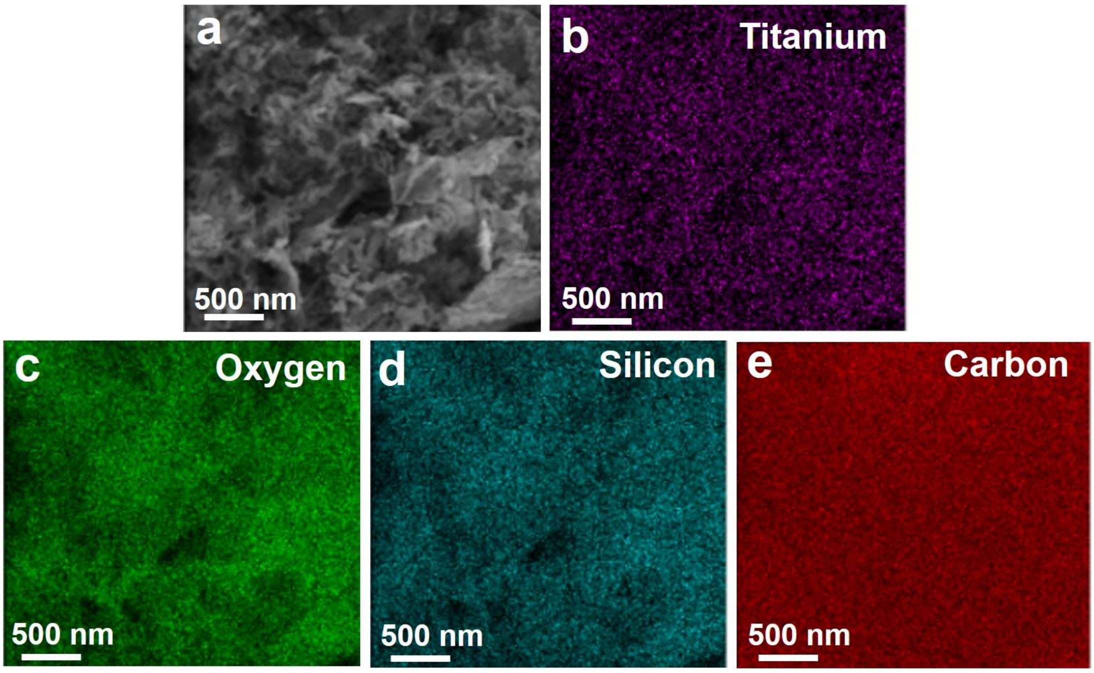

Figure S3. The SEM image (a) of porous MSHA and its corresponding elemental mapping, including Ti (b), O (c), Si (d) and C (e).

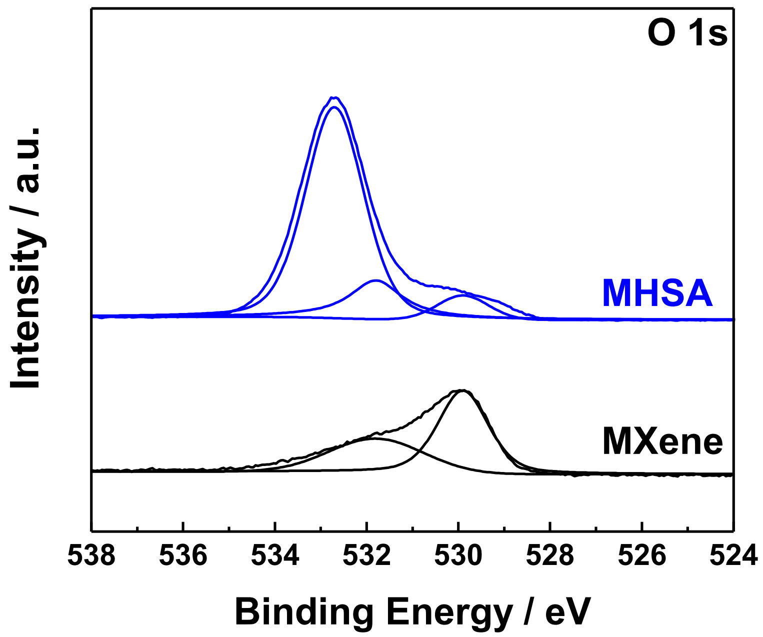

Figure S4. O 1s XPS spectra of MSHA and $Ti_3C_2T_x$ MXene.

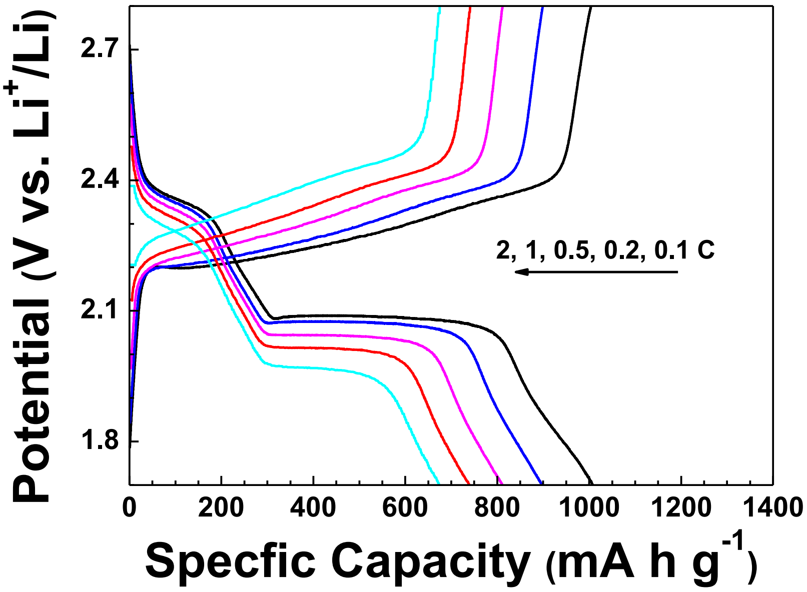

Figure S5. Charge/discharge curves of the cell with the MSHA@S cathode from 0.1 to 2.0 C.

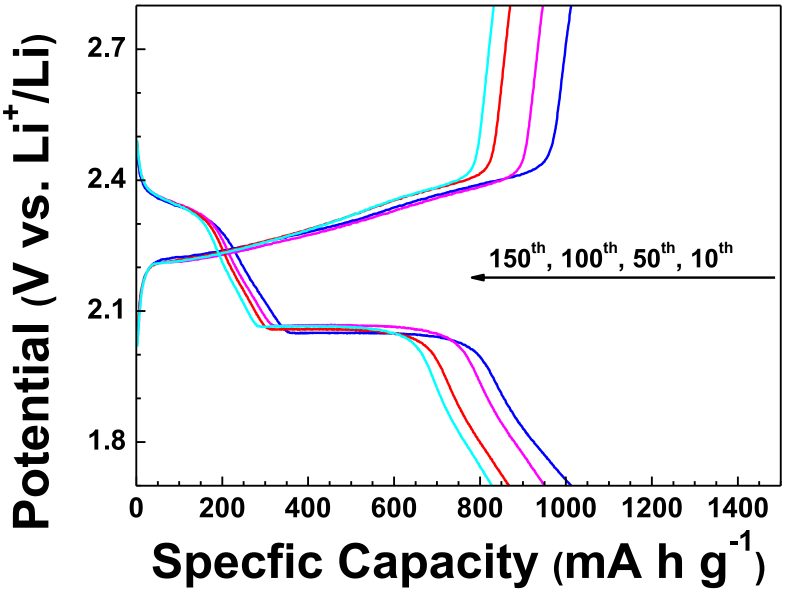

Figure S6. Charge/discharge curves of the cell with the MSHA@S cathode at 0.5 C.

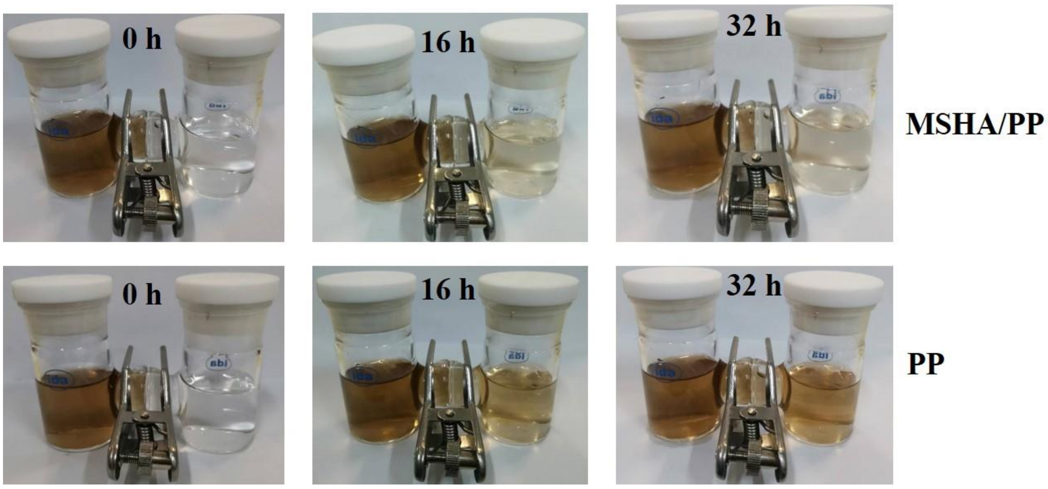

Figure S7. Visualization of the polysulfide diffusion across MSHA/PP and PP separators.

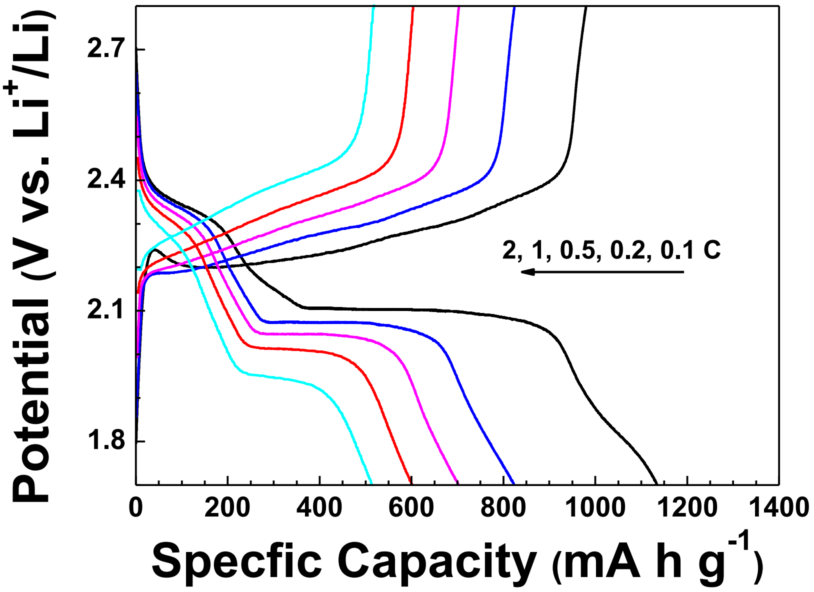

Figure S8. Charge/discharge curves of the cell with MSHA/PP separator from 0.1 to 2.0 C.

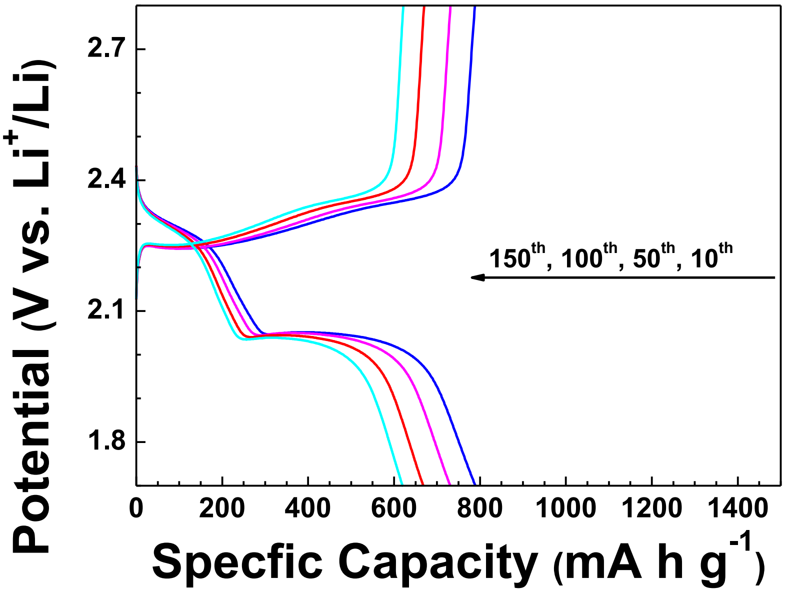

Figure S9. Charge/discharge curves of the cell with MSHA/PP separator at 0.5 C.

References

[1] Lin, L.-W.; Qi, M.; Bai, Z.-T.; Yan, S.-X.; Sui, Z.-Y.; Han, B.-H.; Liu, Y.-W. Crumpled nitrogen-doped aerogels derived from MXene and pyrrole-formaldehyde as modified separators for stable lithium-sulfur batteries. *Appl. Surf. Sci*. **2021**, *555*, 149717.
